# Effects of Photoaged Polyethylene Microplastics on Cardiac Injury and Fibrosis-like Remodeling in Zebrafish

**DOI:** 10.3390/toxics14100889

**Published:** 2026-10-05

**Authors:** Churan Sun, Feifei Yan, Jidong Zhang, Jing Zhang

**Affiliations:** 1 Medical College of Qingdao University, Qingdao 266071, China; 13280985565@163.com; 2 Frontiers Science Center for Deep Ocean Multispheres and Earth System, Key Laboratory of Marine Chemistry Theory and Technology, Ministry of Education, Ocean University of China, Qingdao 266100, China; 3 Institute of Marine Chemistry, Ocean University of China, Qingdao 266100, China; 4 Cardiovascular Internal Medicine Qingdao, The Affliated Hospital of Qingdao University, Qingdao 266071, China; 5 Laboratory for Marine Ecology and Environmental Science, Qingdao Marine Science and Technology Center, Qingdao 266237, China

**Keywords:** polyethylene microplastics, photoaging, zebrafish, cardiotoxicity, extracellular matrix remodeling

## Abstract

Polyethylene microplastics (PE-MPs) undergo photoaging under environmental ultraviolet irradiation, which may alter their surface morphology, chemical properties, and biological toxicity. This study used adult zebrafish to compare cardiac injury induced by pristine and photoaged PE-MPs. Changes in particle physicochemical properties were characterized by scanning electron microscopy and Fourier-transform infrared spectroscopy. Cardiotoxicity was evaluated using organismal phenotypes, cardiac oxidative stress, histopathology, collagen deposition, and related gene expression. Photoaging increased surface roughness, pores, cracks, and oxidation, confirming marked changes in particle structure and chemistry. High-concentration PE-MP exposure inhibited growth. All exposure groups showed decreased cardiac antioxidant enzyme activities and increased lipid peroxidation, with more pronounced oxidative injury in the high-concentration photoaged group. This group also exhibited abnormal myocardial structural disorganization, inflammatory cell infiltration, and fibrosis-like changes. Genes involved in inflammation, profibrotic transcription, and extracellular matrix remodeling were also altered. Overall, PE-MPs induced cardiac oxidative injury, structural abnormalities, and changes associated with collagen remodeling in adult zebrafish, with the combined findings being consistent with fibrosis-like remodeling. Photoaging selectively enhanced some cardiac endpoints, but this effect was not consistently observed across all measured indicators. These findings indicate that environmental aging can modify the cardiotoxicity of PE-MPs and provide experimental evidence for assessing the potential cardiovascular hazards and identifying aging-related changes in toxicity of aged microplastics.

## 1. Introduction

Plastic products can enter aquatic environments, soils, and the atmosphere during their production, use, and disposal. Under the effects of mechanical abrasion, ultraviolet radiation, oxidation, and other environmental processes, plastics gradually fragment into microplastics (MPs), generally defined as plastic particles smaller than 5 mm. Because plastic emissions continue to increase while degradation proceeds slowly, MPs have become widespread in water, sediments, organisms, and food chains, raising growing concerns about their ecological and health implications [1]. Humans can be exposed to MPs through food, drinking water, and inhalation of airborne particles. Plastic particles have been detected in human blood and various tissues, indicating that they can cross certain biological barriers and contribute to internal exposure [2,3,4,5]. Accordingly, research on MPs has gradually expanded from their environmental occurrence and distribution to their accumulation in organisms, associated tissue damage, and potential chronic health effects.

Polyethylene (PE) is one of the most widely produced and used plastics and is also a common polymer type among environmental MPs. After entering natural environments, PE-MPs are continuously exposed to ultraviolet radiation from sunlight, temperature fluctuations, and oxidative conditions, leading to photooxidation characterized by polymer chain scission and the formation of oxygen-containing structures. Laboratory studies frequently use unaged particles with relatively smooth surfaces and uniform composition, whereas PE-MPs in natural environments have generally undergone aging to varying degrees. Therefore, studies based solely on unaged PE-MPs may not adequately represent the actual physicochemical properties and biological effects of environmentally occurring particles. Direct comparisons between unaged and photoaged particles can improve the environmental relevance of microplastic toxicity studies [6,7,8].

Photoaging can increase the surface roughness of PE-MPs, generate pores and cracks, promote surface layer peeling, and facilitate the formation of oxygen-containing functional groups such as carbonyl and hydroxyl groups. These alterations may increase surface polarity and modify particle wettability, aggregation behavior, interfacial reactivity, and interactions with cell membranes and proteins. Polymer oxidation and structural deterioration may also promote the release of low-molecular-weight oxidation products, additives, and other soluble substances. Consequently, biological effects may arise from both the particles themselves and substances released during the aging process [6,7,9,10]. Photoaging may therefore alter the bioavailability, modes of tissue contact, and toxicological characteristics of PE-MPs. However, aging does not necessarily enhance all toxic effects, and its influence may vary depending on polymer type, particle size, degree of aging, exposure medium, and developmental stage of the organism [11,12,13].

The effects of MPs, particularly photoaged MPs, on the human heart remain insufficiently understood. Previous studies have detected MPs in human blood, cardiac-related tissues, arteries, and thrombi. In addition, the presence of microplastics or nanoplastics in atherosclerotic plaques has been associated with an increased risk of subsequent adverse cardiovascular events [2,3,4,5,14]. These findings primarily demonstrate internal exposure and tissue distribution in humans, however, and do not establish a causal relationship between microplastic exposure and cardiovascular disease. Clinical evidence specifically concerning aged MPs is even more limited. Zebrafish share conserved biological processes with mammals in cardiac development, oxidative stress responses, inflammation, and extracellular matrix remodeling, and they are well suited for controlled aquatic exposure and toxicological assessment at multiple biological levels [15,16,17,18]. Nevertheless, zebrafish possess a two-chambered heart and a strong capacity for myocardial regeneration, and concentrations used in aquatic exposure experiments cannot be directly converted into internal exposure doses in humans. Thus, this model is primarily suitable for identifying potential hazards and generating mechanistic hypotheses rather than directly predicting clinical outcomes in humans [19,20,21].

Previous studies in fish and zebrafish have shown that exposure to PE-MPs or aged MPs can alter antioxidant enzyme activity, promote lipid peroxidation, disrupt the expression of inflammation-related genes, and impair mitochondrial and tissue functions [6,7,9,22,23,24,25,26]. In zebrafish, MP exposure has also been associated with reduced heart rate, abnormal cardiac development, myocardial tissue injury, and inflammatory responses [22,23,27]. However, most existing studies have focused on embryos or larvae, or have evaluated only unaged particles or a limited set of toxicological endpoints. Direct comparisons of the cardiac effects of unaged and photoaged PE-MPs in adult zebrafish remain limited. Whether photoaging aggravates cardiac oxidative injury, inflammatory alterations, and collagen deposition, and whether these changes collectively reflect remodeling with fibrosis-like features, have yet to be systematically investigated [28].

Environmental monitoring has shown that PE is among the predominant microplastic polymers in aquatic environments, although reported concentrations vary considerably depending on sampling location, the lower particle size detection limit, and the method used for quantification. For example, microplastic abundance in the surface water of Nainital Lake, India, ranged from 8.6 to 56.0 particles/L, with high-density polyethylene identified as the predominant polymer. In the Hunter River in Australia, microplastic abundance ranged from 47 to 229 particles/L, and PE was likewise among the dominant polymer types [29,30]. A study of Taihu Lake using mass-based quantification further reported concentrations of PE microplastics and nanoplastics smaller than 100 μm ranging from 20.3 to 635.7 μg/L, equivalent to 0.0203–0.6357 mg/L [31]. In comparison, the nominal concentrations of 5 and 25 mg/L used in the present study were approximately 7.9 and 39.3 times, respectively, the upper limit of the measured PE concentration reported in that study.

Therefore, under controlled experimental conditions, the present study used two nominal concentrations, 5 and 25 mg/L, to compare the effects of unaged and photoaged PE-MPs on general phenotypic responses, cardiac oxidative stress, tissue morphology, collagen deposition, and associated transcriptional responses in adult zebrafish. Particular attention was given to whether photoaging alters the cardiotoxicity profile of PE-MPs and whether the resulting changes are consistent with inflammation-related responses and extracellular matrix remodeling with fibrosis-like features. These exposure concentrations were selected for comparative hazard identification rather than to simulate typical exposure levels in natural aquatic environments.

## 2. Materials and Methods

### 2.1. Preparation of Polyethylene Microplastic Exposure Suspensions and Photoaging Treatment

Polyethylene (PE) microplastics used in this study were purchased from Teshulang Chemical Materials Co., Ltd. (Dongguan, China). According to the manufacturer, the particles had a nominal diameter of approximately 50 μm, were white and spherical with a relatively uniform size distribution, contained no fluorescent labeling, and had a purity greater than 99%. Photoaging was conducted in a custom-built ultraviolet aging chamber with controlled temperature and humidity. The system consisted of an independent chamber for temperature and humidity control and UVB 313 nm fluorescent ultraviolet lamps (Dongguan Chuangu Lighting Technology Co., Ltd., Dongguan, China), which served as the ultraviolet radiation source. PE-MPs were placed in the chamber in a dry state within a fixed sample irradiation area and were aged under the same exposure geometry. To minimize contamination by environmental dust, fibers, and other exogenous materials during prolonged dry aging, the treatment was performed in a relatively enclosed irradiation chamber. Before each experiment, the interior of the chamber and the sample holding area were thoroughly cleaned. Clean utensils were used during sample preparation and transfer, and the time for which the samples were exposed to the open laboratory environment was minimized. Throughout the aging period, all treatment groups were handled using identical containers and procedures to reduce systematic bias arising from external contamination or operational differences. Based on the light output parameters provided by the lamp manufacturer and the lamp configuration used in the experimental system, the nominal irradiance at the sample area was estimated to be approximately 10 mW/cm^2^. This value represents a calculated nominal irradiance derived from the manufacturer specifications rather than an independent measurement obtained at the sample plane using a calibrated irradiance meter. The chamber temperature was maintained at 28 ± 2 °C, with relative humidity controlled at 50 ± 5%, and the PE-MPs were continuously treated for 28 d. Unaged PE-MPs were processed in parallel using the same containers, temperature, humidity, treatment duration, and other handling conditions but without exposure to UVB radiation. This treatment was designed to produce laboratory-accelerated aging of PE-MPs with evident surface photooxidative characteristics rather than to quantitatively reproduce environmental aging under natural sunlight. The UV-treated particles were designated as photoaged PE microplastics (Aged-MPs), whereas particles not exposed to UV radiation were designated as unaged PE microplastics (U-MPs).

After completion of the photoaging treatment, U-MPs and Aged-MPs were separately rinsed three times with deionized water and then dried at 40 °C for 24 h. This procedure was intended to reduce the influence of loosely attached surface materials and residual soluble low-molecular-weight compounds on the subsequent exposure experiments. Mild treatment conditions were used throughout this process. Strong acids, strong alkalis, organic solvents, and high temperatures that could induce thermal degradation of PE were avoided. The purpose of this step was to standardize the condition of the two particle types before biological exposure rather than to induce further material aging. U-MPs and Aged-MPs were subsequently suspended separately in distilled water and dispersed by water-bath ultrasonication using a digitally controlled ultrasonic cleaner. The instrument had a nominal operating frequency of 40 kHz and a rated ultrasonic power of 300 W. The power setting was adjusted to 80% of the rated output, and ultrasonication was performed for 30 min. The primary purpose of ultrasonication was to promote uniform dispersion of PE-MPs in the aqueous phase and reduce visible particle aggregation rather than to intentionally alter particle size or surface properties. After ultrasonication, the resulting particle suspensions were serially diluted using the same aqueous medium to prepare PE-MP exposure suspensions at nominal concentrations of 5 and 25 mg/L for the formal exposure experiments. The preparation of the exposure suspensions was adapted from previous studies of PE microplastic exposure in adult zebrafish, with appropriate modifications based on the experimental conditions used here. The photoaging procedure and the parallel comparison with pristine PE microplastics were designed with reference to previous studies comparing aged and pristine PE particles [7,22].

### 2.2. Physicochemical Characterization of Microplastics

U-MPs and Aged-MPs were characterized using scanning electron microscopy (SEM) and Fourier transform infrared spectroscopy (FTIR) to evaluate changes in surface morphology and functional groups before and after photoaging. The analytical framework was based on previous studies examining morphological and chemical structural changes in aged PE microplastics [7]. SEM imaging was performed using a SEM5000X instrument (CIQTEK, China) equipped with an ETD SE detector. Samples were coated with gold (Au) prior to imaging. Images were acquired at a magnification of 6000×, a dwell time of 3 μs, and a scale bar of 2 μm. The accelerating voltage and working distance were 5 kV and 10.04 mm for U-MPs, and 10 kV and 9.63 mm for Aged-MPs, respectively. Because the accelerating voltages differed between the two groups, SEM images were used only for qualitative comparison of morphological features, including surface cracks, pores, and lamellar peeling. Parameters such as crack depth were not quantitatively evaluated from the two-dimensional images. FTIR analysis was performed using a Thermo Fisher Scientific Nicolet iS20 Fourier transform infrared spectrometer. The original spectra were recorded over a range of approximately 500–4000 cm^−1^ at a spectral resolution of 4 cm^−1^, with 32 scans accumulated for each sample. The original FTIR spectra were expressed as transmittance, T (%). To facilitate comparison of the relative intensities of characteristic absorption bands before and after photoaging, transmittance was converted to absorbance according to the equation A = −log_10_(T/100). Characteristic PE absorption bands near 2913, 2847, 1468, and 716 cm^−1^, together with absorption features near 1722, 3409, and 1169 cm^−1^ associated with oxygen-containing functional groups, were analyzed to evaluate changes in the surface chemical structure of PE-MPs induced by photoaging. SEM and FTIR were used in this study to verify whether UV treatment produced detectable surface morphological and chemical features associated with photooxidation, rather than to provide a comprehensive characterization of all colloidal and interfacial properties of the aged particles. Therefore, the present study does not infer particle size distribution, specific surface area, zeta potential, surface charge, wettability, aggregation, or sedimentation behavior from the available data.

### 2.3. Experimental Animals and Husbandry Conditions

Healthy six-month-old male AB strain zebrafish (Danio rerio) were used in this study. The fish were maintained in continuously aerated E3 medium filtered through a 0.45 μm membrane under a 14 h light and 10 h dark photoperiod. Artemia cysts were provided twice daily. The formal exposure experiment was conducted in independent tanks with a nominal capacity of 10 L. Each tank contained 8 L of exposure medium and 60 adult zebrafish, corresponding to an initial stocking density of 7.5 fish/L. Before the experiment, the independent tanks were randomly assigned to the different treatment groups at the tank level. During the exposure period, each treatment group consisted of separate exposure tanks, with each independent biological replicate corresponding to one individual tank. Husbandry and medium renewal were performed separately for each tank, and neither fish nor exposure medium was mixed among replicate tanks. The culture medium and corresponding treatment solutions were renewed daily to maintain consistent exposure conditions across groups. To minimize potential systematic bias associated with fixed spatial positions, the locations of the tanks were periodically exchanged during the exposure period. Zebrafish husbandry and care were performed according to established recommendations for zebrafish maintenance [32], and the design and reporting of the animal experiments were guided by the ARRIVE 2.0 guidelines [33]. Information on the zebrafish supplier, the composition of the E3 medium, and specific water quality parameters is provided in the Supplementary Table S1.

### 2.4. Acute Toxicity Test

A concentration gradient of U-MPs was established, consisting of a control group and exposure groups at 400, 800, 1200, 1600, and 2000 mg/L. Each group included three independent biological replicates, with 10 zebrafish in each replicate at the start of the experiment. The acute toxicity test was conducted in independent tanks with a nominal capacity of 10 L. Each tank contained 8 L of exposure medium. Except for the concentration of PE-MPs, all groups were maintained under identical conditions, including the culture medium, temperature, photoperiod, and other experimental parameters. Zebrafish survival was assessed at the start of exposure and after 8, 16, 24, 48, 72, and 96 h. Mortality was determined using a caudal fin stimulation test. Fish that showed no movement within 5 s after gentle stimulation of the caudal fin were considered dead [34]. Newly occurring deaths were recorded at each observation time point, and cumulative mortality rates were calculated for each group. A probit model was subsequently used to establish the concentration–response relationship. The acute toxicity test in this study was conducted using only unaged PE-MPs (U-MPs). Its purpose was to establish the short-term concentration–response range of the parent PE particles and to provide an initial reference for selecting concentrations for subsequent sublethal exposure, rather than to compare the acute lethal toxicity of unaged and photoaged particles.

### 2.5. Exposure Protocol, Biological Replication, and Cardiac Tissue Collection

The main exposure experiment consisted of five groups: the control group (Ctrl), the 5 mg/L pristine PE-MP group (U-MPs-L), the 25 mg/L pristine PE-MP group (U-MPs-H), the 5 mg/L photoaged PE-MP group (Aged-MPs-L), and the 25 mg/L photoaged PE-MP group (Aged-MPs-H). For each treatment group, 180 adult zebrafish were distributed among three independent exposure tanks, with 60 fish per tank. Because PE-MP treatment was applied at the tank level, the tank was defined as the experimental unit for treatment, resulting in three independent tanks for each treatment group (n = 3). Exposure was conducted under semistatic conditions, with the exposure medium renewed daily. Samples were collected on days 7 and 28 to assess biological responses at different stages of exposure. At each sampling time point, zebrafish were allocated according to the predefined experimental design for phenotypic, biochemical, histological, and RNA analyses. For cardiac biochemical and RNA analyses, hearts were dissected on ice within 5 min after euthanasia. The isolated hearts were gently rinsed with precooled phosphate-buffered saline until no visible blood remained, blotted dry with filter paper, and processed immediately. No mortality occurred in any group during the 28 d exposure period.

### 2.6. Body Length and Body Weight

On day 28 of exposure, feeding was withheld for 24 h in all exposure tanks before sampling. Ten zebrafish were randomly selected from each independent tank in each treatment group for measurements of body length and body weight. The measurements obtained from the 10 fish within each tank were first averaged, and the mean values from the three independent tanks were then used for statistical comparisons among groups. Therefore, the statistical sample size for these endpoints was n = 3. Day 28 was selected to evaluate relatively stable biological effects after sustained exposure and to maintain consistency with the sampling time point used for subsequent histopathological examination and assessment of collagen deposition. To minimize the effects of spontaneous body movement on morphometric measurements, zebrafish were briefly anesthetized in 0.016% tricaine solution (160 mg/L) preequilibrated to 28 °C. Timing began when each fish was placed in the anesthetic solution, and morphometric measurements were initiated immediately after 1.5 min of anesthesia. Standard body length was measured from the tip of the snout to the base of the caudal peduncle using a digital caliper (Mitutoyo 500-196-30, Kawasaki, Japan; accuracy, 0.01 mm). Excess surface water was then gently removed using clean absorbent material, and body weight was measured with an analytical balance (Mettler Toledo ME204, Tokyo, Japan; accuracy, 0.1 mg). The same anesthetic concentration, temperature, and exposure duration were used for all treatment groups to ensure consistency in comparisons among groups.

### 2.7. Biochemical Indicators Related to Cardiac Oxidative Stress Measurements

On day 7, cardiac total superoxide dismutase (T-SOD) activity, catalase (CAT) activity, and malondialdehyde (MDA) content were measured, whereas only T-SOD activity was assessed on day 28. Twelve hearts were collected from each independent exposure tank and pooled within that tank to generate one tissue homogenate. Thus, at each time point, each treatment group yielded three independent pooled samples derived from three separate tanks (n = 3 tanks). The 12 hearts included in each pooled sample were considered subsamples within the same tank and were not treated as 12 independent biological replicates. The two sampling time points were selected to characterize oxidative stress responses at different stages of exposure. Day 7 was used to assess early stress responses, particularly changes in antioxidant defense and lipid peroxidation, whereas day 28 was used to further evaluate alterations in antioxidant capacity after sustained exposure based on T-SOD activity. After collection, the hearts were gently rinsed with precooled PBS, and excess surface liquid was carefully removed. For each pooled sample, precooled 0.01 mol/L PBS (pH 7.4) was added at a tissue mass to homogenization buffer volume ratio of 1:9 (*w*/*v*). The samples were homogenized under low-temperature conditions using a high-speed tissue grinder (Servicebio, SWE-FP, Wuhan, China) at 60 Hz for 60 s, followed by a 30 s interval, for a total of two cycles. The homogenates were then centrifuged at 10,000× *g* for 10 min at 4 °C. After homogenization and centrifugation, the supernatants were collected for measurement of the corresponding biochemical parameters and protein concentration.

After homogenization of cardiac tissue, T-SOD, CAT, MDA, and BCA protein assay kits from Servicebio^®^ were used according to the manufacturer’s instructions. T-SOD activity was determined using the WST-1 method, with absorbance measured at 450 nm; CAT activity was assessed using the ammonium molybdate colorimetric method at 405 nm; and MDA content was determined using the thiobarbituric acid (TBA) method at 532 nm. Absorbance was measured using a BioTek Epoch (Santa Clara, CA, USA) microplate reader. Protein concentrations were determined using the BCA method and used to normalize enzyme activities, with the colorimetric principle consistent with the classical assay method [35]. T-SOD, CAT, and MDA were selected as indicators of cardiac oxidative stress in zebrafish based on previous studies of microplastic-induced cardiac toxicity [22,36].

### 2.8. Hematoxylin and Eosin Staining and Qualitative Histomorphological Evaluation of Cardiac Tissue

On day 28, cardiac tissues were collected from each group. Each treatment group consisted of three independent exposure tanks, and three zebrafish were randomly selected from each tank for HE histological evaluation. Collected hearts were fixed in 4% paraformaldehyde for 24 h, followed by dehydration, paraffin embedding, and preparation of serial sections at a thickness of 4 μm. The sections were subsequently subjected to hematoxylin and eosin (HE) staining and mounted for microscopic examination. Histomorphological evaluation of adult zebrafish hearts was conducted with reference to previous studies of microplastic-induced cardiac injury [22,27]. To reduce potential bias associated with evaluating a single tissue level, three nonconsecutive sections separated by 30 μm were selected from each zebrafish for HE staining. The stained sections were scanned using a Servicebio LG-S80 digital pathology slide scanner and examined in Saiviewer software 2.1.1 (Servicebio, China) at 10× and 40× magnification. HE staining was used for descriptive histomorphological assessment, with particular attention to myocardial tissue integrity, cardiomyocyte arrangement and cytoplasmic morphology, interstitial eosinophilic material, localized erythrocyte aggregation, and inflammatory cell infiltration. All sections included in the evaluation underwent systematic descriptive examination. However, no semiquantitative histopathological severity scoring system was established a priori, and no quantitative data on lesion incidence or severity scores were generated for statistical analysis. Therefore, HE findings were treated solely as a qualitative and supportive histomorphological endpoint and were not used for inferential statistical comparisons of lesion severity among treatment groups. The microscopic images presented in the manuscript were selected to illustrate representative morphological features observed during the systematic examination and were not used to estimate lesion incidence or severity.

### 2.9. Sirius Red and Masson Staining and Quantification of Collagen-Positive Area

Using the procedures described above, paraffin sections of hearts collected from each group on day 28 were subjected to Sirius Red staining and Masson trichrome staining to evaluate collagen deposition and changes associated with extracellular matrix remodeling in cardiac tissue. The use of these two staining methods to identify collagen and assess collagen deposition and extracellular-matrix-remodeling-related changes in the heart was based on established Sirius Red staining protocols and previous studies of microplastic-related cardiac fibrosis [37,38]. Quantification of myocardial collagen area in zebrafish was additionally performed with reference to a previously reported method [22]. Digital sections were analyzed using Aipathwell^®^ software 2.1.1 (Servicebio, China). Regions of interest (ROIs) were first delineated along the boundaries of the cardiac tissue, and collagen-positive areas were identified according to staining color characteristics in the HSI color space. The automatically identified regions were manually reviewed when necessary to exclude obvious nontissue areas or misclassified regions. The Aipathwell^®^ software 2.1.1 (Servicebio, China) then automatically calculated the positively stained area and the total tissue area. For both Sirius Red and Masson staining, the percentage of collagen-positive area was calculated as the positively stained area divided by the total tissue area and multiplied by 100%. Results from multiple sections obtained from the same fish were first summarized at the individual animal level. The animal-level results within each independent tank were then aggregated, and the independent tank was used as the statistical unit for comparisons among treatment groups (n = 3). An increase in collagen-positive area alone was not considered an independent diagnostic criterion for myocardial fibrosis. Instead, collagen deposition was interpreted together with HE histomorphological observations and transcriptional changes in extracellular matrix and profibrotic-related genes to evaluate tissue alterations consistent with fibrosis-like remodeling.

### 2.10. Total RNA Extraction and Reverse Transcription Measurements

At the designated sampling time points, 15 zebrafish were randomly selected from each independent exposure tank in each treatment group. Following the same brief anesthesia procedure described above, the zebrafish were euthanized according to the experimental protocol, and the hearts were rapidly dissected. The 15 hearts collected from each independent tank were pooled to generate one RNA sample, with no pooling across tanks. Thus, each treatment group ultimately yielded three independent tank-derived RNA biological replicates (n = 3). Each biological replicate was homogenized at low temperature in 1 mL of RNA extraction reagent (Servicebio, G3013) using three 3 mm grinding beads. The homogenate was centrifuged at 13,700× *g* for 10 min at 4 °C. The supernatant was mixed with 100 μL of chloroform substitute (Servicebio, G3014) and allowed to stand for 3 min. After a second centrifugation, 400 μL of the upper aqueous phase was transferred to a new tube, mixed with 550 μL of isopropanol, and incubated at −20 °C for 15 min to precipitate RNA. The resulting RNA pellet was washed twice with 1 mL of 75% ethanol, briefly air-dried, and dissolved in 15 μL of RNA dissolution solution. RNA concentration and purity were determined using a NanoDrop 2000 spectrophotometer (Wilmington, DE, USA), and samples with higher RNA concentrations were diluted to 200 ng/μL. Reverse transcription was performed using SweScript All-in-One RT SuperMix for qPCR with One-Step gDNA Remover (Servicebio, G3337). The 20 μL reaction mixture contained 4 μL of 5× SuperMix, 1 μL of gDNA Remover, 10 μL of total RNA, and nuclease-free water to a final volume of 20 μL. The reverse transcription program consisted of incubation at 25 °C for 5 min, 42 °C for 30 min, and 85 °C for 5 s. Experimental procedures and reporting were documented in accordance with the MIQE guidelines [39].

### 2.11. Quantitative Real-Time PCR and Relative Gene Expression Measurements

Quantitative real-time PCR (qPCR) was performed using a Bio-Rad CFX Connect system (Hercules, CA, USA). Each 15 μL reaction contained 7.5 μL of 2× Universal Blue SYBR Green qPCR Master Mix (Servicebio, G3326), 1.5 μL of a 2.5 μM forward and reverse primer mixture, 2.0 μL of cDNA, and 4.0 μL of nuclease-free water. The amplification program consisted of an initial denaturation at 95 °C for 30 s, followed by 40 cycles of 95 °C for 15 s and 60 °C for 30 s. After amplification, melting curve analysis was performed from 65 to 95 °C, with fluorescence signals recorded at 0.5 °C increments. The presence of a single dominant peak in the melting curve was used as quality control evidence supporting the relative specificity of the amplification product, but was not considered the sole criterion for confirming the identity of the target product. The target genes included il1b, acta2, ccn2a, and col1a1a on days 7 and 28, and mmp9 on day 28. eef1a1l1 (EF1A) was used as the reference gene. Each cDNA sample was analyzed in three technical replicates. The mean Ct value of the technical replicates was first calculated, and statistical analyses were then performed using three independent biological replicates.

For the ith biological replicate, the relative expression level of the target gene was calculated using the 2−ΔΔCt method [40]. The calculation formulas were as follows:

(1) $$\Delta Ct_{i}=Ct_{t\arg et_{i}}-Ct_{eflA}$$

(2) $$\Delta \Delta Ct_{i}=\Delta Ct_{i}-mean\Delta Ct_{Ctrl}$$

In Equations (1) and (2), $Ct_{t\arg et_{i}}$ represents the Ct value of the target gene in the ith biological replicate; $Ct_{eflA}$ represents the Ct value of the reference gene eflA in the i-th biological replicate; $\Delta Ct_{i}$ represents the difference between the Ct value of the target gene and that of the reference gene in the ith biological replicate; $mean\Delta Ct_{Ctrl}$ represents the arithmetic mean of the $\Delta Ct_{i}$ values for the corresponding target gene in the Ctrl group at the same time point; and 2−ΔΔCt represents the relative expression level of the target gene in the ith biological replicate. To normalize the arithmetic mean of the relative expression levels in the Ctrl group to 1, the relative expression value of each sample was further divided by the arithmetic mean of the relative expression values in the Ctrl group at the same time point. All calculations were performed at the level of independent biological replicates. Technical replicates were averaged before calculation and were not treated as independent statistical replicates. The primer sequences used for quantitative PCR are listed in Supplementary Table S2.

### 2.12. Statistical Analysis

All statistical analyses were performed using GraphPad Prism 10.1.2. Data meeting the assumptions for parametric testing were expressed as the mean ± standard deviation (mean ± SD), with individual biological replicate values also displayed. The independent exposure tank was defined as the experimental unit in the formal exposure experiment. Each treatment group consisted of three independent exposure tanks; therefore, unless otherwise specified, the sample size for inferential statistical comparisons among groups was n = 3. Multiple zebrafish, hearts, tissue sections, fields of view, and technical replicates obtained from the same tank were treated as subsamples or technical replicates within that experimental unit and were not considered additional independent n values. For comparisons among the five groups at a single time point, the assumptions for parametric analyses were evaluated primarily by inspecting Q–Q plots of model residuals, with the Shapiro–Wilk test used as a supplementary assessment of residual distribution. Homogeneity of variance was assessed using the Brown–Forsythe test. For comparisons among the five groups at a single time point, ordinary one-way analysis of variance was used when the data satisfied the assumptions of normality and homogeneity of variance, followed by Tukey’s multiple comparisons test. When the data were approximately normally distributed but showed unequal variances, Welch’s one-way analysis of variance was applied together with the corresponding multiple comparisons procedure. For data showing a clear deviation from normality, the Kruskal–Wallis test followed by Dunn’s multiple comparisons test was used. All statistical tests were two-sided, and *p* < 0.05 was considered statistically significant.

## 3. Results

### 3.1. Acute Toxicity and Concentration and Time Effects of PE-MP Exposure

**Figure 1:**
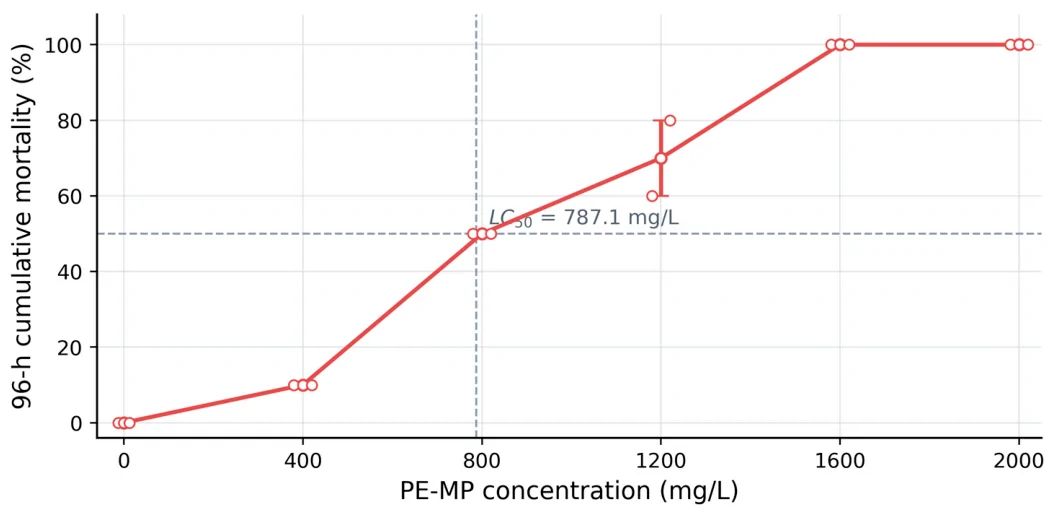
Mean cumulative mortality of zebrafish after 96 h exposure to different concentrations of PE-MPs (n = 3).

The 96 h acute toxicity test showed no mortality in the control group, whereas cumulative mortality increased with increasing U-MP concentration, demonstrating a clear concentration-dependent response. The mean cumulative mortality rates were 10%, 50%, and 70% in the 400, 800, and 1200 mg/L groups, respectively, and reached 100% in both the 1600 and 2000 mg/L groups. Mortality also occurred earlier at higher exposure concentrations, with all zebrafish in the 2000 mg/L group dying within 16 h and those in the 1600 mg/L group dying within 48 h. Probit analysis yielded a 96 h LC_50_ of 787.1 mg/L. As shown in Figure 1 and Table 1, the formal 28 d experiment used nominal laboratory exposure concentrations of 5 and 25 mg/L to establish low and high exposure levels and to compare the relative effects of U-MPs and Aged-MPs. For descriptive reference, these concentrations corresponded to approximately 0.64% and 3.18%, respectively, of the 96 h LC_50_ for U-MPs. These percentages were not used as a basis for determining the suitability of the concentrations for long-term exposure or their environmental relevance. The selected concentrations were intended to establish an experimental exposure gradient for comparative hazard assessment and were not considered representative of typical exposure levels in natural aquatic environments.

### 3.2. Effects of Photoaging on the Surface Morphology and Chemical Properties of PE-MPs

**Figure 2:**
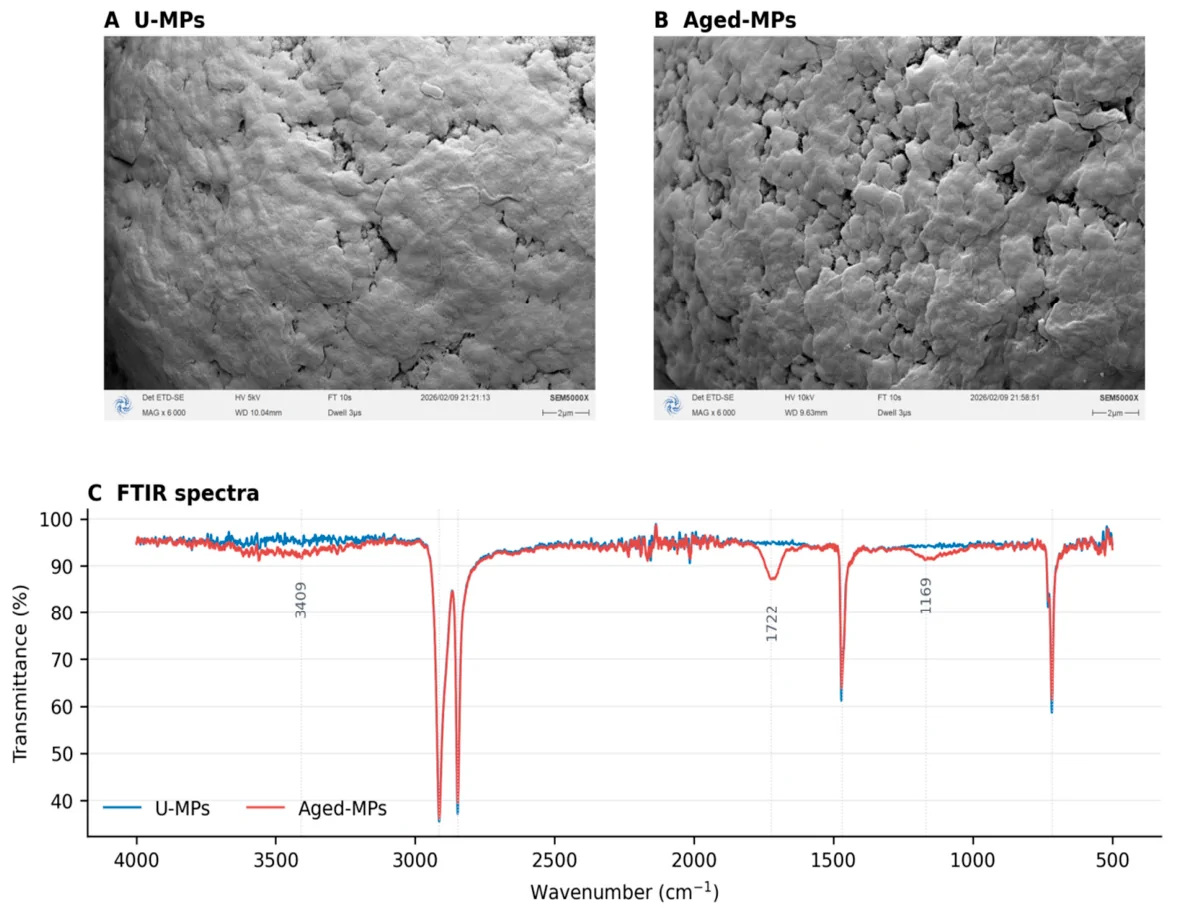
Surface morphology and FTIR spectra of PE-MPs before and after photoaging. (**A**) U-MPs; (**B**) aged MPs; (**C**) FTIR transmittance spectra.

At a magnification of 6000× with a scale bar of 2 μm, the surface of U-MPs appeared relatively continuous and compact, with only a few shallow surface cracks. In contrast, Aged-MPs exhibited a markedly rougher and looser surface, with more extensive lamellar peeling, pores, and visible cracks. Because the SEM images of U-MPs and Aged-MPs were acquired at accelerating voltages of 5 and 10 kV, respectively, these images were used only for qualitative comparison of surface morphology. As shown in Figure 2A,B, no quantitative inference regarding three-dimensional structural parameters, such as crack depth, was made from the SEM images.

FTIR spectra showed that both U-MPs and Aged-MPs retained the characteristic PE absorption bands near 2913, 2847, 1468, and 716 cm^−1^. After photoaging, the absorption intensities near 1722, 3409, and 1169 cm^−1^ increased, indicating an increase in spectral features associated with oxygen-containing functional groups on the PE-MP surface. After transmittance was converted to absorbance using A = −log_10_(T/100), the apparent carbonyl index, A_1722_/A_1468_, increased from 0.133 to 0.330, corresponding to an increase of 148.4%. The apparent hydroxyl index, A_3409_/A_1468_, increased from 0.140 to 0.198, corresponding to an increase of 42.2%. These changes are illustrated in Figure 2C and summarized in Table 2. The substantially greater increase in the carbonyl index than in the hydroxyl index indicates that ultraviolet irradiation enhanced the oxygen-containing chemical features of PE-MPs, particularly those associated with carbonyl structures, consistent with a pronounced photooxidation process. Taken together, the SEM and FTIR results show that 28 d of ultraviolet irradiation produced concurrent changes in the surface morphology and chemical oxidation state of PE-MPs, characterized by surface roughening, increased cracking and porosity, and enhanced oxidation-related spectral features. These physicochemical alterations provide a material basis for the subsequent comparison of biological effects between pristine and photoaged PE-MPs.

### 3.3. Effects of PE-MP Exposure on Zebrafish Growth

**Figure 3:**
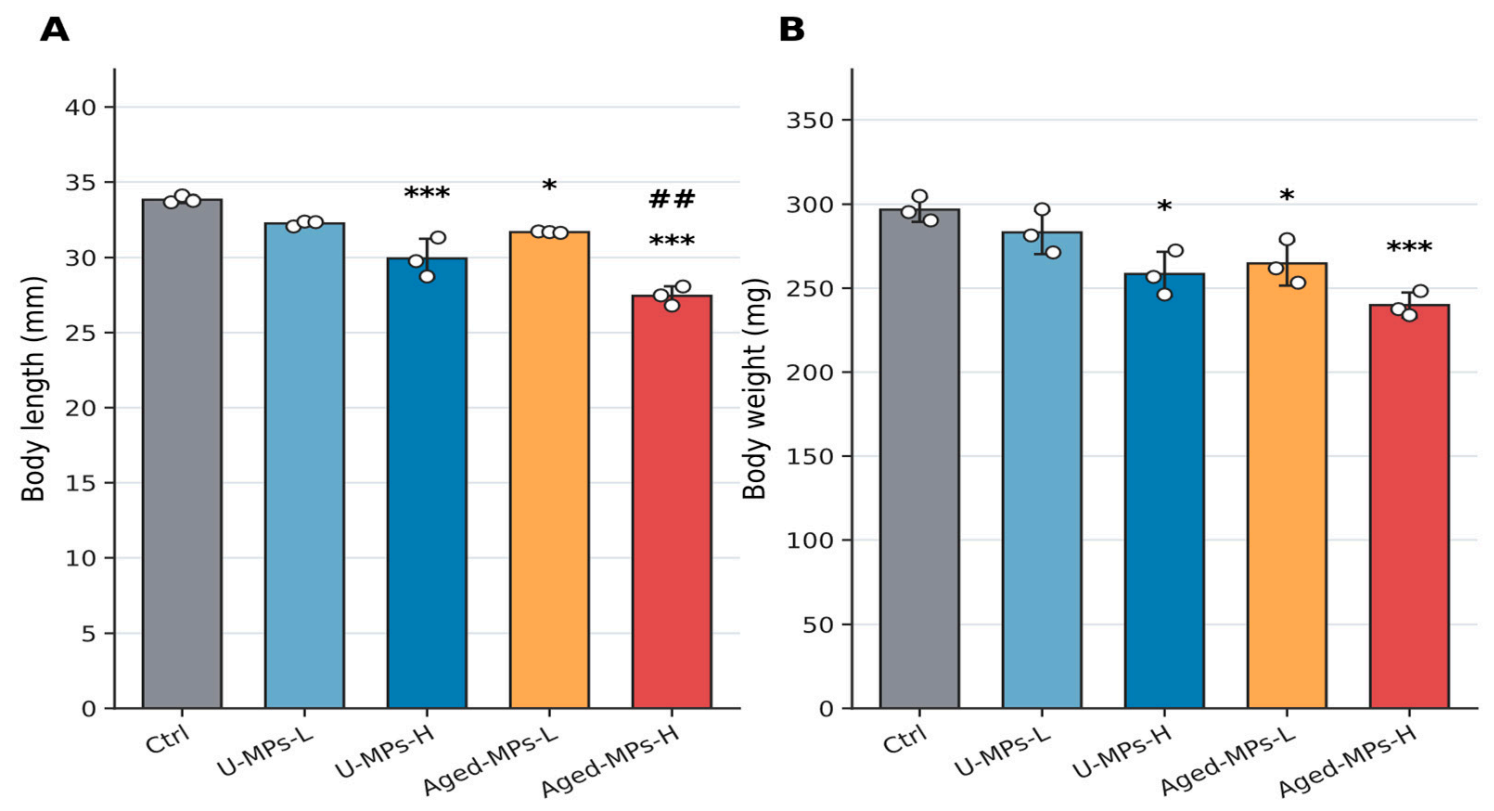
Body length and body weight of adult zebrafish after 28 d of exposure to pristine or photoaged PE-MPs. (**A**) Body length. (**B**) Body weight. Bars represent the mean ± SD (n = 3), and open circles indicate individual tank means. * *p* < 0.05 and *** *p* < 0.001 versus the Ctrl group; ## *p* < 0.01 versus the U-MPs-H group.

After 28 d of exposure, analyses based on three biological-replicate means per group (n = 3; 10 fish per replicate) showed significant treatment effects on body length [F(4,10) = 40.91, *p* < 0.0001] and body weight [F(4,10) = 11.67, *p* = 0.0009] (Figure 3). Compared with the Ctrl group, body length and body weight were significantly reduced in the U-MPs-H, Aged-MPs-L, and Aged-MPs-H groups (*p* < 0.05). At matched concentrations, body length was significantly lower in the Aged-MPs-H group than in the U-MPs-H group (*p* = 0.0067), whereas no significant difference was observed between the two low-concentration groups. Body weight did not differ significantly between the corresponding unaged and photoaged groups at either concentration (*p* > 0.05). These findings indicate that PE-MP exposure affected growth-related phenotypes, whereas the additional effect associated with photoaging was endpoint-specific.

### 3.4. Effects of Photoaging on Cardiac Oxidative Stress Induced by PE-MPs in Zebrafish

Statistical analyses of cardiac biochemical indicators were based on three independent biological replicates per group. By day 7, PE-MP exposure had already induced marked oxidative stress in the heart. Compared with the Ctrl group, T-SOD activity decreased by 14.3% to 39.2% across all exposure groups (all *p* < 0.001), with the greatest reduction observed in the Aged-MPs-H group. T-SOD activity in this group was further reduced by 20.4% relative to the U-MPs-H group (*p* < 0.001). In parallel, MDA content increased by 169.9% to 563.9% across the exposure groups, indicating a substantial increase in lipid peroxidation. Compared with the corresponding U-MP groups, MDA levels were further elevated by 52.4% in the Aged-MPs-L group and 44.4% in the Aged-MPs-H group (*p* = 0.0015 and *p* < 0.001, respectively), showing that photoaging enhanced MDA accumulation at both exposure concentrations. CAT activity decreased by 39.0% to 51.2% across all exposure groups compared with the Ctrl group (all *p* < 0.001). However, CAT activity in the Aged-MPs-H group was 25.0% higher than that in the U-MPs-H group (*p* = 0.0144), indicating a response pattern that was not fully consistent with the changes observed in T-SOD activity and MDA content. By day 28, T-SOD activity remained markedly suppressed, with reductions of 68.5% to 78.8% relative to the Ctrl group (all *p* < 0.001). T-SOD activity in the Aged-MPs-H group was also 21.9% lower than that in the U-MPs-H group (*p* = 0.0047). These findings indicate that suppression of cardiac antioxidant defense persisted during sustained exposure and was more pronounced in the high-concentration photoaged group.

**Figure 4:**
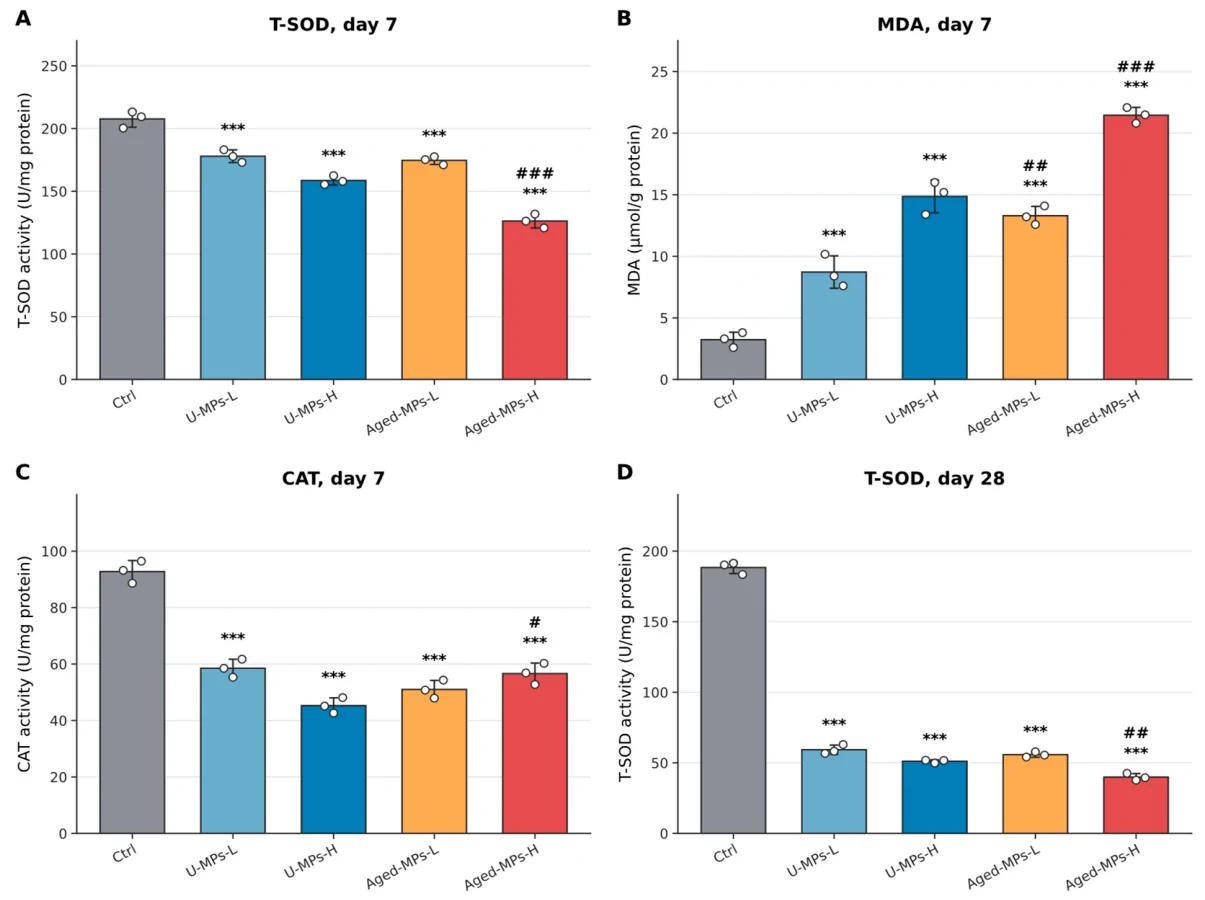
Cardiac oxidative-stress indicators after PE-MP exposure: T-SOD activity on day 7 (**A**), MDA content on day 7 (**B**), CAT activity on day 7 (**C**), and T-SOD activity on day 28 (**D**). Data are presented as mean ± SD (n = 3); circles show individual biological replicates. *** *p* < 0.001 versus the Ctrl group; # *p* < 0.05, ## *p* < 0.01, and ### *p* < 0.001 versus the concentration-matched U-MPs group.

Overall, PE-MP exposure impaired cardiac antioxidant defense and enhanced lipid peroxidation in zebrafish, indicating a marked disruption of cardiac redox homeostasis. The influence of photoaging was mainly reflected by a further reduction in T-SOD activity and a further increase in MDA content, whereas CAT activity did not follow the same response pattern. Taken together with the results presented in Figure 4, these findings indicate that photoaging enhanced selected cardiac oxidative stress responses, although the effect was not consistent across all oxidative stress indicators.

### 3.5. Histomorphological Changes in Zebrafish Hearts Following PE-MP Exposure

**Figure 5:**
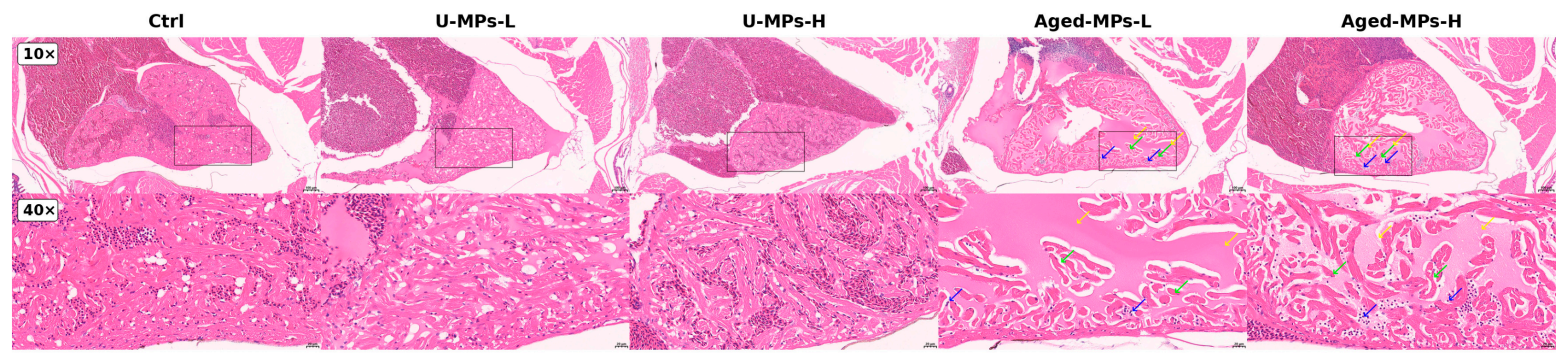
Representative HE-stained sections of zebrafish hearts after 28 d of PE-MP exposure. Columns correspond to the Ctrl, U-MPs-L, U-MPs-H, Aged-MPs-L, and Aged-MPs-H groups. Upper and lower rows show 10× and 40× views, respectively. Scale bars are 100 μm (10×) and 20 μm (40×). Green arrows indicate reduced cardiomyocyte size, yellow arrows indicate eosinophilic exudates, and blue arrows indicate inflammatory cell infiltration.

Systematic descriptive examination of the tissue sections used for HE evaluation showed that, after 28 d of exposure, myocardial architecture in the Ctrl group remained generally intact, with a relatively clear epicardial boundary, regularly arranged cardiomyocytes, and relatively uniform staining. No obvious abnormal histomorphological features were observed. In some sections from the U-MPs-L and U-MPs-H groups, localized loosening of cardiomyocyte arrangement, narrowing of some cardiomyocyte profiles or loose-appearing cytoplasmic changes, and erythrocyte aggregation in interstitial or vascular regions were observed. In some sections from the Aged-MPs-L group, abnormal cardiomyocyte arrangement, narrowing of some cardiomyocyte profiles or apparent reduction in cytoplasmic volume, interstitial eosinophilic material, and inflammatory cell-like infiltration were observed. Similar types of histomorphological alterations were also observed in the Aged-MPs-H group, including abnormal cardiomyocyte arrangement, altered cytoplasmic morphology, interstitial eosinophilic material, and inflammatory cell-like infiltration. Figure 5 presents representative histomorphological features observed during the systematic examination of sections from each treatment group. Overall, myocardial histomorphological alterations were observed after prolonged PE-MP exposure. Abnormal features observed in the photoaged PE-MP groups included changes in cardiomyocyte morphology, interstitial eosinophilic material, and inflammatory cell-like infiltration. These HE findings were interpreted as supportive histological evidence in conjunction with the other study endpoints.

### 3.6. Effects of Photoaging on Myocardial Collagen Deposition Induced by PE-MPs in Zebrafish

Collagen staining on day 28 showed varying degrees of myocardial collagen deposition after PE-MP exposure, with more pronounced changes in the photoaged groups. Sirius Red staining showed mean collagen-positive area ratios of 22.38%, 22.51%, 24.06%, 30.01%, and 47.60% in the Ctrl, U-MPs-L, U-MPs-H, Aged-MPs-L, and Aged-MPs-H groups, respectively. Compared with the Ctrl group, the collagen-positive area increased by 34.1% in the Aged-MPs-L group and by 112.7% in the Aged-MPs-H group. Compared with the corresponding U-MP groups, the increases were 33.3% and 97.8%, respectively, with the greatest collagen deposition observed in the Aged-MPs-H group. The Masson staining results were generally consistent with those obtained using Sirius Red staining. The mean collagen-positive area ratios were 20.73%, 21.83%, 25.80%, 29.45%, and 36.12% in the five groups, respectively. Relative to the Ctrl group, the values increased by 42.1% and 74.3% in the Aged-MPs-L and Aged-MPs-H groups, respectively. Compared with the corresponding pristine PE-MP groups, the increases were 34.9% and 40.0%, respectively. The results of the two staining methods were analyzed separately using one-way analysis of variance followed by Tukey multiple comparisons. Significant overall differences among groups were detected for both Sirius Red staining [F(4,10) = 99.59, *p* < 0.0001] and Masson trichrome staining [F(4,10) = 45.04, *p* < 0.0001].

**Figure 6:**
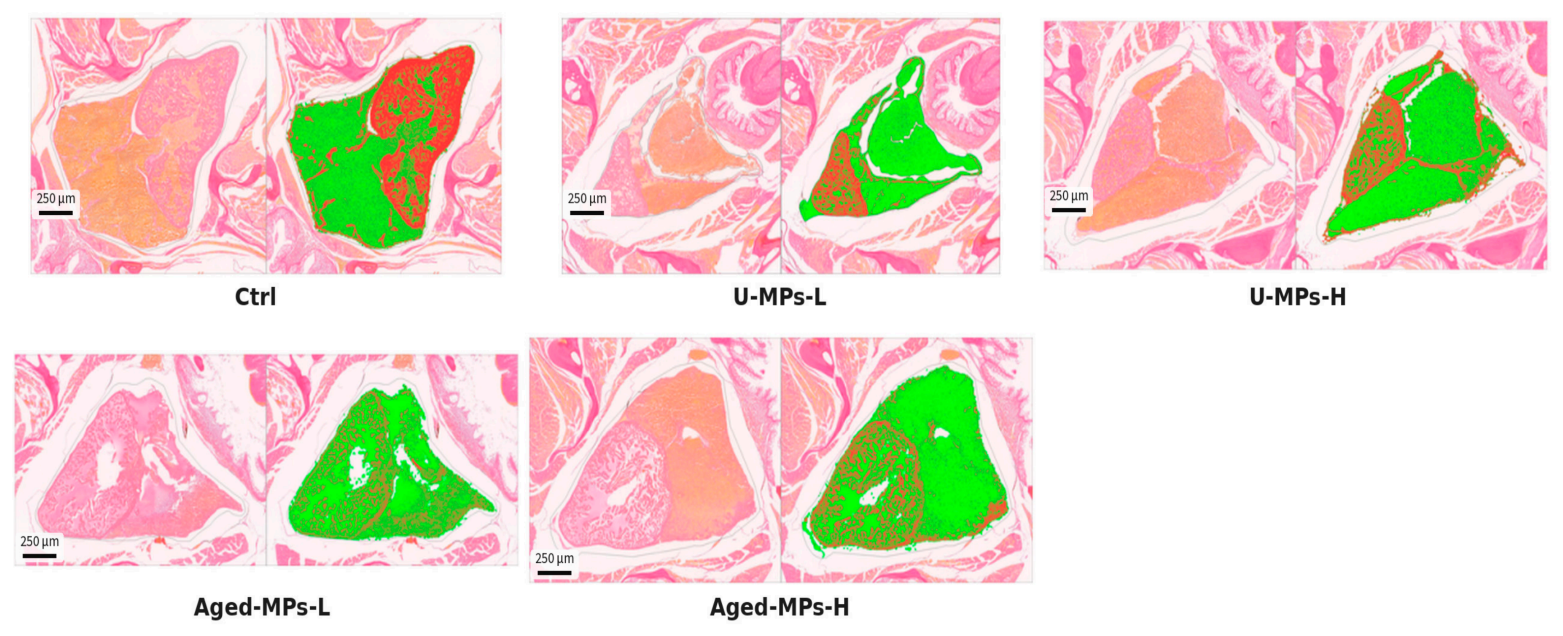
Representative Sirius Red-stained zebrafish heart sections and the corresponding segmentation overlays after 28 d of PE-MP exposure. Within each group, the original image is shown on the left and the software-generated segmentation overlay on the right. Green denotes the analyzed tissue region and red denotes collagen-positive staining within that region. Scale bars = 250 μm; segmentation overlays are displayed at the same scale as the paired original images.

**Figure 7:**
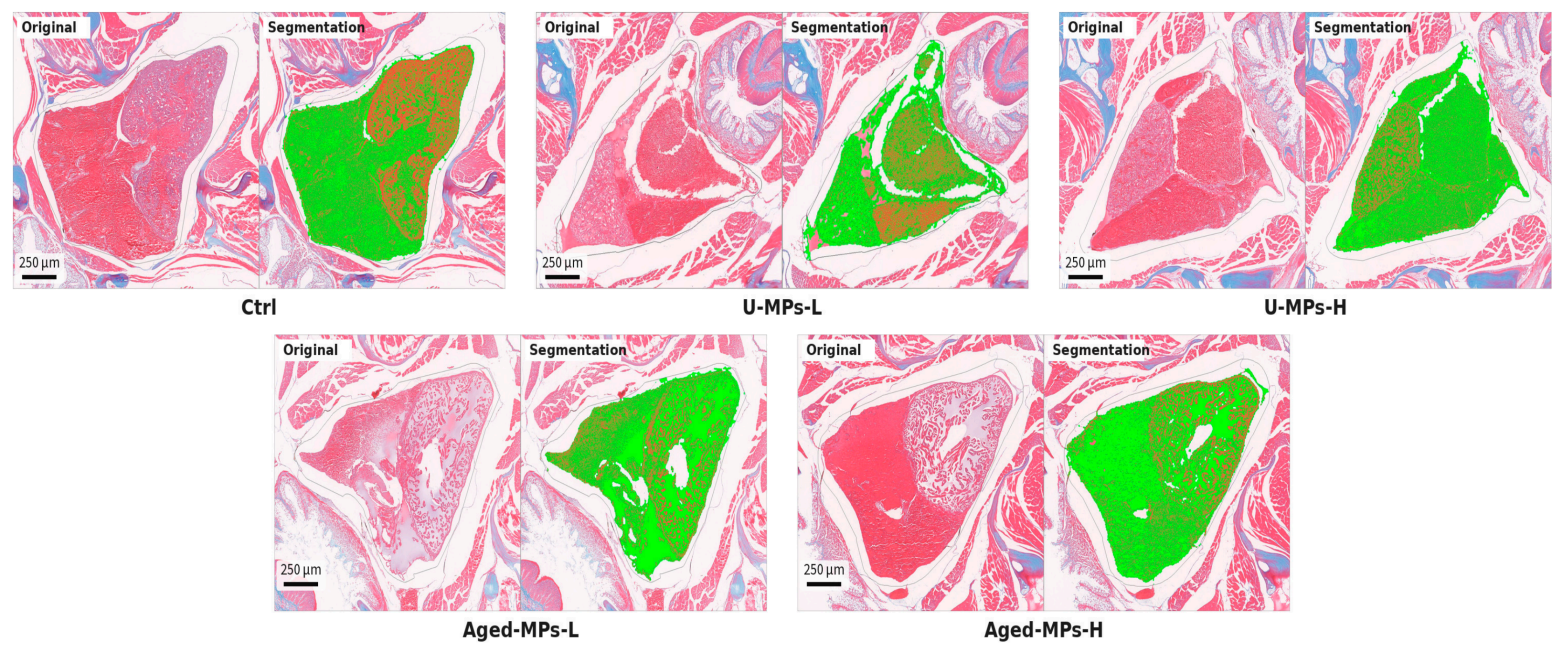
Representative Masson-stained zebrafish heart sections and the corresponding segmentation over-lays after 28 d of PE-MP exposure. Within each group, the original image is shown on the left and the software-generated segmentation overlay on the right. Green denotes the analyzed tissue region and red denotes collagen-positive staining within that region. Scale bars = 250 μm; segmentation overlays are displayed at the same scale as the paired original images.

**Figure 8:**
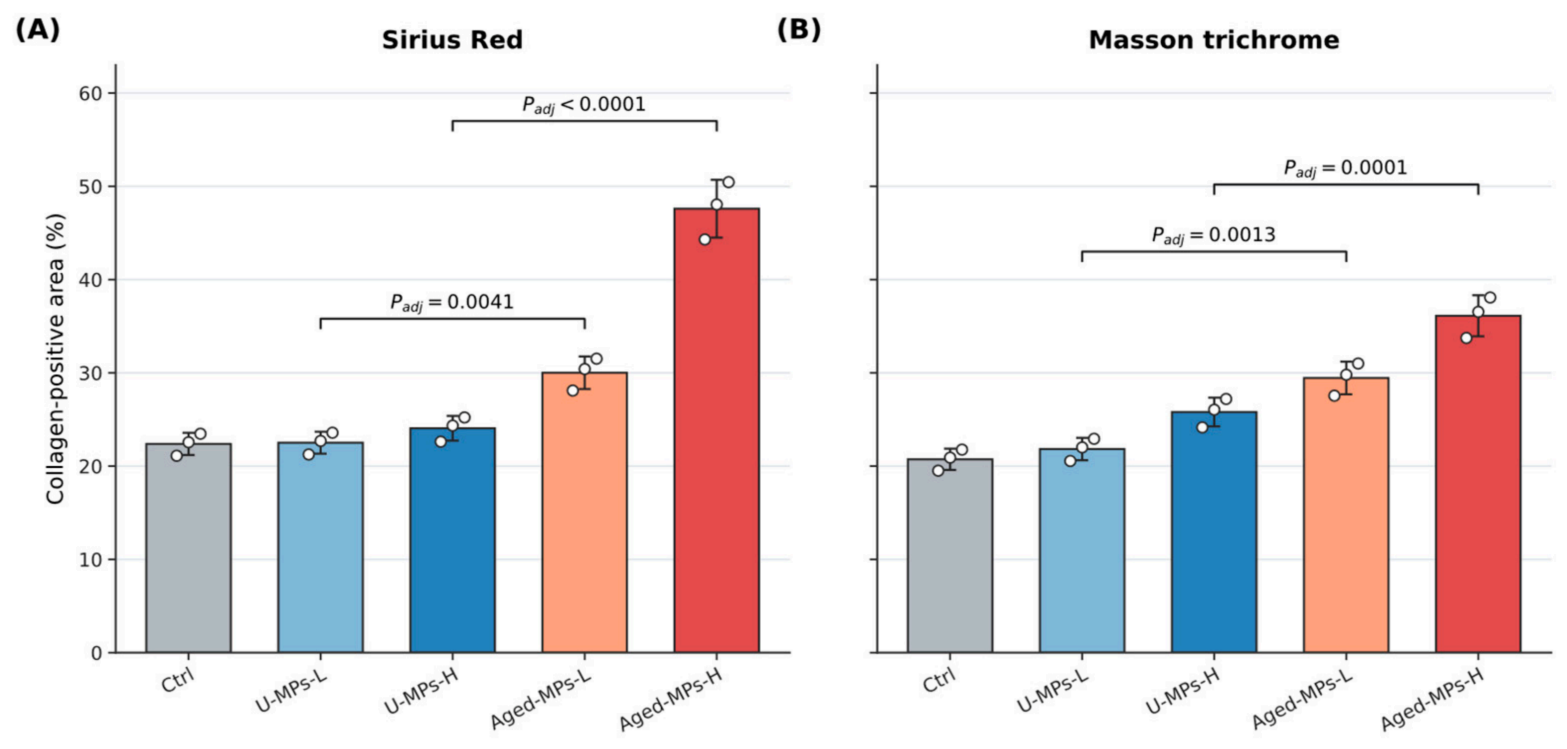
Quantification of collagen-positive area in adult zebrafish hearts after 28 d of PE-MP exposure. (**A**) Sirius Red staining; (**B**) Masson trichrome staining. Bars represent the mean ± SD, and individual points represent independent biological replicates (n = 3 per group).

Overall, both independent histochemical staining methods showed changes in the proportion of collagen-positive area in the heart following PE-MP exposure, with higher mean collagen-positive area percentages in the Aged-MP groups than in the corresponding U-MP groups at the same exposure concentrations. Where the corresponding Tukey pairwise comparisons reached statistical significance, these findings support an association between photoaging and enhanced cardiac collagen deposition induced by PE-MPs. When interpreted together with the HE histomorphological observations and transcriptional changes in ECM and profibrotic-related genes, these results support alterations consistent with fibrosis-like extracellular matrix remodeling (Figure 6, Figure 7 and Figure 8 and Table 3).

### 3.7. Effects of Photoaging on Inflammation and Stress-Related Gene Expression Induced by PE-MPs in Zebrafish Hearts

**Figure 9:**
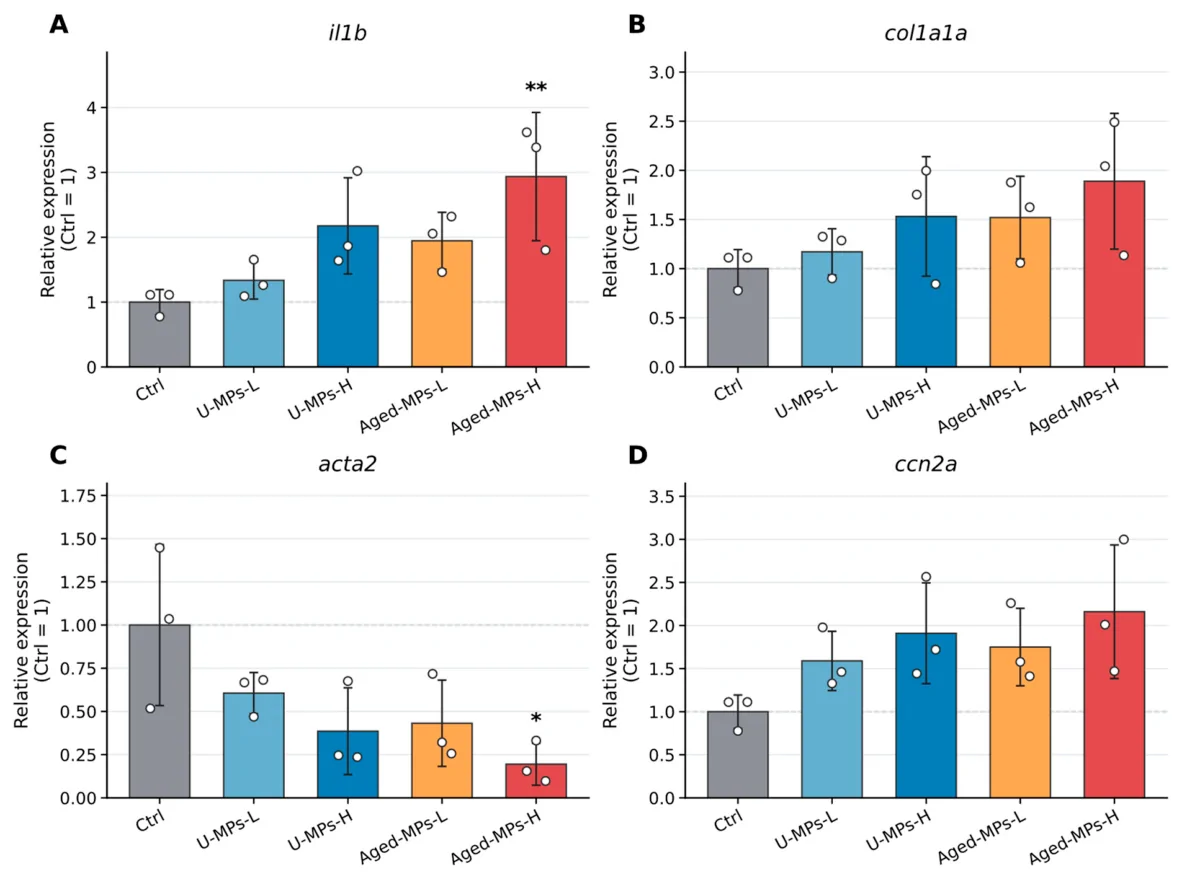
Relative cardiac gene expression after 7 d of PE-MP exposure: il1b (**A**), col1a1a (**B**), acta2 (**C**), and ccn2a (**D**). Data are presented as mean ± SD (n = 3); circles show individual biological replicates. Statistical testing was performed using ΔCt values. * *p* < 0.05 and ** *p* < 0.01 versus the Ctrl group.

On day 7, qPCR analysis showed significant overall group differences in il1b and acta2 expression [F(4,10) = 6.34, *p* = 0.0083 and F(4,10) = 4.43, *p* = 0.0256, respectively; Figure 9]. Compared with the Ctrl group, the relative expression of il1b in the Aged-MPs-H group increased by 193.5% (Tukey, *p* = 0.0074), whereas acta2 expression decreased by 80.5% (*p* = 0.0177). The relative expression levels of ccn2a and col1a1a in the Aged-MPs-H group increased by 116.0% and 88.9%, respectively, but the overall group effects did not reach statistical significance (*p* = 0.0533 and *p* = 0.2906, respectively).

Overall, the most clearly supported molecular changes on day 7 were the upregulation of il1b and the downregulation of acta2, whereas ccn2a and col1a1a showed only numerical increases. These findings indicate that, at the earlier stage of exposure, the cardiac molecular response to photoaged PE-MPs was characterized primarily by enhanced inflammation-related signaling and stress-associated transcriptional changes, while genes related to profibrotic processes and collagen synthesis did not yet show consistent or statistically significant alterations.

### 3.8. ECM Remodeling and Profibrotic-Related Gene Expression

**Figure 10:**
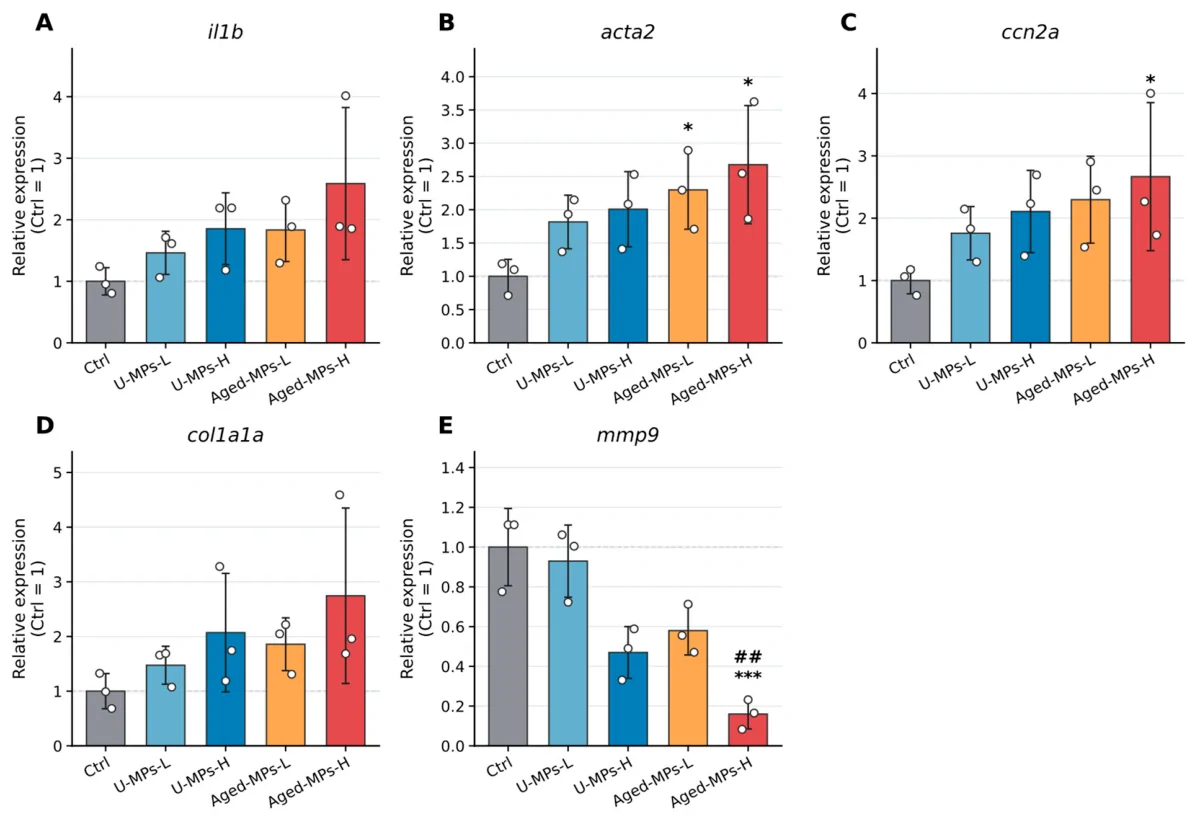
Relative cardiac gene expression after 28 d of PE-MP exposure: il1b (**A**), acta2 (**B**), ccn2a (**C**), col1a1a (**D**), and mmp9 (**E**). Data are presented as mean ± SD (n = 3); circles show individual biological replicates. Statistical testing was performed using ΔCt values. * *p* < 0.05 and *** *p* < 0.001 versus the Ctrl group; ## *p* < 0.01 versus the U-MPs-H group.

On day 28, significant overall group differences were observed in the expression of acta2, ccn2a, and mmp9 [F(4,10) = 5.23, *p* = 0.0155; F(4,10) = 3.82, *p* = 0.0389; and F(4,10) = 17.98, *p* < 0.001, respectively; Figure 10]. Compared with the Ctrl group, the relative expression of acta2 increased by 129.9% in the Aged-MPs-L group and by 167.7% in the Aged-MPs-H group (*p* = 0.0308 and *p* = 0.0123, respectively). The relative expression of ccn2a increased by 166.7% in the Aged-MPs-H group (*p* = 0.0343). In contrast, mmp9 expression decreased by 84.0% in the Aged-MPs-H group compared with the Ctrl group (*p* < 0.001) and was further reduced by 66.0% relative to the U-MPs-H group (*p* = 0.0087). The relative expression levels of col1a1a and il1b in the Aged-MPs-H group increased by 174.4% and 158.8%, respectively. However, the overall group differences did not reach statistical significance (*p* = 0.1306 and *p* = 0.0635, respectively), and these changes should therefore be interpreted only as increasing trends.

Overall, the molecular changes observed on day 28 were characterized mainly by significant upregulation of acta2 and ccn2a together with marked downregulation of mmp9. These transcriptional changes were consistent with the concurrent histological findings of increased myocardial collagen deposition, suggesting disrupted extracellular matrix homeostasis and profibrotic molecular responses after prolonged exposure and supporting the occurrence of fibrosis-like remodeling. In contrast, the changes in col1a1a and il1b did not provide statistically conclusive evidence.

## 4. Discussion

In this study, adult zebrafish were used as an experimental model to compare the cardiac effects of unaged and photoaged polyethylene microplastics (PE-MPs) at the same nominal concentrations. Material characterization showed that 28 d of UV irradiation increased surface roughness, pores, and cracks on PE-MPs and enhanced spectral features associated with oxygen-containing functional groups. In terms of biological effects, PE-MP exposure was associated with alterations in growth, impaired cardiac antioxidant defense, increased lipid peroxidation, myocardial histomorphological abnormalities, and greater collagen deposition. The high-concentration photoaged group showed larger changes in several cardiac-related endpoints. This overall pattern is broadly consistent with previous studies on the cardiovascular toxicity of PE or PS microplastics and the biological effects of aged microplastics [7,9,22,27]. However, photoaging did not produce an independently detectable enhancement across all endpoints. At the same exposure concentration, the effects associated with photoaging were endpoint-specific. Among the general phenotypic comparisons at equivalent concentrations, only body length in the Aged-MPs-H group was significantly lower than that in the U-MPs-H group; body weight did not differ significantly. Body length also did not differ significantly between the two low-concentration groups. Therefore, the present findings support an association between photoaging and enhancement of selected cardiotoxicity endpoints rather than a general increase in all toxic effects of PE-MPs.

Changes in particle surface properties may represent an initial factor contributing to the observed differences in toxicity. The surface layer peeling, increased roughness, and greater abundance of cracks observed by SEM were consistent with enhanced FTIR absorption near 1722, 3409, and 1169 cm^−1^, together with increases in the apparent carbonyl and hydroxyl indices. These findings indicate that 28 d of UV irradiation induced surface oxidation and structural deterioration while largely retaining the characteristic PE backbone. Similar changes have been reported in studies of aged PE and other polymer microplastics [6,7,9,10]. Mechanistically, increases in surface defects and oxygen-containing groups may alter particle wettability, aggregation behavior, and interactions with cell membranes, proteins, and coexisting contaminants, thereby modifying biological reactivity. However, the present study did not measure particle size distribution, specific surface area, zeta potential, contact angle, aggregation or sedimentation kinetics in the exposure medium, additives, or soluble photooxidation products. In addition, UV irradiance was based on a nominal value rather than an independently measured value at the sample plane. Consequently, the observed cardiac injury cannot be attributed directly to any single surface property, nor can the accelerated aging procedure be quantitatively equated with the time course of photoaging under natural environmental conditions. Although unaged particles were subjected to dark control treatment under the same temperature, humidity, and handling conditions, and aged particles were washed and dried before subsequent exposure, no control corresponding to the soluble fraction or leachate from Aged-MPs was included. The current design therefore primarily reflects the overall effects of washed photoaged particles and cannot further distinguish the relative contributions of altered particle surface properties from those of soluble substances released during exposure. Accordingly, differences between U-MPs and Aged-MPs should be interpreted as differences in the overall effects of the two particle preparations under the present exposure system rather than as evidence that a specific surface property directly caused the observed toxicity differences. Previous work has also shown that photooxidation can reduce the developmental toxicity of polyamide microplastics, further indicating that the biological consequences of aging depend on multiple factors, including polymer type, particle size, exposure medium, and developmental stage [10]. Future studies combining more detailed characterization of particle surface properties with analysis of soluble products generated during aging may help distinguish the respective contributions of changes in the particles themselves and aging-derived soluble products [41].

After 28 d of exposure, reductions in body length or body weight were observed in some high-concentration PE-MP groups, consistent with the direction of growth inhibition changes reported in previous zebrafish microplastic studies [22,23,27,34]. However, no significant differences in body weight were detected between unaged and photoaged particles at the same concentration, indicating that any additional effect of photoaging on these general phenotypes was limited or below the detection capacity of the present experiment. Body length and body weight may also be influenced by food intake, activity, and whole-body metabolic status. These endpoints are therefore useful as indicative measures of general toxicity changes but should not be used independently to infer mechanisms of myocardial injury. In particular, changes in these variables alone cannot be attributed to fibrosis-like remodeling.

Cardiac redox imbalance was one of the major biological processes examined in this study. On day 7, T-SOD and CAT activities were reduced and MDA accumulation was increased across the exposed groups, while the Aged-MPs-H group showed greater lipid peroxidation and lower T-SOD activity. Suppression of T-SOD activity remained evident on day 28. Previous studies in adult zebrafish, other fish species, and zebrafish hearts have similarly shown that exposure to PE-MPs, naturally weathered microplastics, or other microplastics can be accompanied by altered antioxidant defense, lipid peroxidation, and myocardial injury [7,22,24,25,27]. Together, these findings support the involvement of impaired antioxidant defense and enhanced lipid peroxidation in PE-MP-related cardiotoxicity. Notably, CAT activity on day 7 was higher in the Aged-MPs-H group than in the U-MPs-H group, which was not fully consistent with the patterns observed for T-SOD and MDA. This discrepancy indicates that antioxidant responses were not uniform across endpoints. It may reflect compensatory regulation, redistribution of antioxidant enzyme activity, or other nonmonotonic responses, although additional mechanistic evidence would be required to distinguish among these possibilities. The influence of photoaging on oxidative stress should therefore be interpreted on an endpoint-specific basis. Because ROS levels were not measured directly and no antioxidant intervention was performed, the present study cannot establish oxidative stress as the causal initiating event leading to subsequent tissue injury [42]. The temporal assessment of cardiac oxidative stress in this study has several limitations. Owing to the limited amount of cardiac tissue available for biochemical assays and resource constraints, T-SOD, CAT, and MDA were measured on day 7, whereas only T-SOD was assessed on day 28. Therefore, only T-SOD could be evaluated at both time points. The CAT and MDA results reflect early antioxidant defense and lipid peroxidation responses and cannot be used to infer their persistence, recovery, or further deterioration on day 28. Future studies should include larger independent sample sizes and consistent panels of biochemical endpoints across all time points to provide a more complete assessment of the temporal progression of cardiac oxidative injury.

Inflammation-related transcriptional changes and histomorphological abnormalities occurred concurrently in this study, providing support for a possible association among cardiac injury responses at different biological levels. On day 7, il1b was significantly upregulated in the Aged-MPs-H group, suggesting that an inflammation-related transcriptional response had already emerged during the early exposure period. By day 28, representative HE sections showed altered cardiomyocyte arrangement and cytoplasmic morphology, interstitial eosinophilic material, and inflammatory cell-like infiltration. Previous studies in adult zebrafish have reported changes in immune-related gene expression after microplastic exposure, while cardiac toxicity studies have also documented the coexistence of oxidative stress, inflammatory responses, and tissue injury [22,26,27]. Nevertheless, the HE results in the present study were derived from systematic descriptive examination without a predefined blinded semiquantitative scoring system, and neither inflammatory infiltration nor myocardial structural abnormalities were subjected to inferential statistical analysis. The HE findings should therefore be regarded as supportive morphological evidence and cannot establish that tissue injury was statistically more severe in one treatment group than in another. Taken together, the biochemical, transcriptional, and histological findings support a working hypothesis in which redox imbalance and inflammatory responses may jointly contribute to cardiac injury, but the sequential causal relationship between these processes remains to be tested through intervention studies.

The collagen staining and gene expression results obtained on day 28 collectively indicated extracellular matrix remodeling. Both Sirius Red and Masson staining revealed significant overall differences in collagen-positive area among groups. In direct comparisons based on mean values from independent tanks, collagen-positive area was greater in the photoaged groups than in the corresponding unaged groups at the same concentrations, with the highest value observed in the Aged-MPs-H group. At the same time, acta2 expression was increased in both the Aged-MPs-L and Aged-MPs-H groups, while ccn2a was increased and mmp9 was decreased in the Aged-MPs-H group. In contrast, although col1a1a showed an increasing trend, the overall difference among groups did not reach statistical significance. This combination of findings can be interpreted as increased collagen deposition accompanied by profibrotic transcriptional responses and altered matrix turnover, rather than as evidence that all fibrosis-related genes were significantly upregulated. Previous animal studies have suggested that microplastic-related myocardial remodeling may involve Wnt/β-catenin or ROS-TGF-β1/Smad signaling and may be accompanied by changes in α-SMA and CCN2-related responses [37,43,44]. However, the present study did not measure TGF-β1, phosphorylation of Smad2/3, β-catenin, collagen protein abundance, fibroblast-specific markers, or spatial colocalization. Thus, although the current findings are compatible with increased expression of factors related to the TGF-β/Smad-CCN2 axis and Wnt/β-catenin signaling, they do not demonstrate activation of either pathway. The available evidence supports an enhanced profibrotic phenotype and transcriptional changes related to fibrotic remodeling and is consistent with fibrosis-like extracellular matrix remodeling. Confirmation of the underlying mechanisms will require protein-level validation and pathway-specific intervention.

Changes in mmp9 expression further suggest that extracellular matrix remodeling cannot be explained solely by increased collagen synthesis. On day 28, mmp9 expression was significantly reduced in the Aged-MPs-H group and was also lower than that in the U-MPs-H group at the same concentration. During zebrafish cardiac injury and regeneration, mmp9 is involved in inflammatory cell recruitment, extracellular matrix degradation, and scar resolution, while matrix composition changes dynamically across different stages of tissue repair [45,46]. The downregulation of acta2 on day 7 followed by its upregulation on day 28 also suggests that early stress or disruption of contractile structures and later reparative remodeling may exhibit distinct transcriptional characteristics. On this basis, a conceptual working framework can be proposed that links material surface aging, redox imbalance, inflammation-related alterations, and extracellular matrix remodeling. The value of this model lies in integrating biological responses across multiple levels rather than treating their temporal sequence as direct evidence of causality. Accordingly, mmp9 may participate in both early injury and inflammatory responses and later collagen turnover, with its biological consequences depending on exposure stage, the cellular source of MMP9, and the overall balance of the MMP/TIMP regulatory network. However, samples collected on days 7 and 28 were independent and did not represent longitudinal observations of the same animals. In addition, MMP9 enzymatic activity, TIMP levels, and candidate pathway proteins were not measured, and no pathway-blocking or rescue experiments were performed. This working model therefore serves to integrate multiple levels of evidence and should not be interpreted as proof that temporal continuity or a causal pathway has been established.

The exposure concentrations and acute toxicity findings should be interpreted within the limits of environmental relevance. The concentrations of 5 and 25 mg/L were substantially higher than most environmental monitoring levels summarized in the Introduction. Using the highest reported mass concentration of 0.6357 mg/L as a descriptive reference, these experimental concentrations were approximately 7.9 and 39.3 times higher, respectively, although particle size ranges, sampling methods, and measurement units are not fully comparable across studies. The 96 h acute toxicity test was performed only with unaged PE-MPs. The resulting LC_50_ and the corresponding proportions of 0.64% and 3.18% were used only to guide the selection of sublethal experimental concentrations that avoided obvious mortality. These values do not demonstrate environmental realism of the selected concentrations and cannot be used to infer that photoaged particles have the same acute toxicity. Furthermore, nominal rather than measured concentrations were used in the present study, and neither particle recovery nor time-weighted actual concentrations was determined. Under conditions without continuous mixing and with daily medium renewal, sedimentation, aggregation, adsorption to tank walls, and removal during medium replacement may all alter the actual particle concentration in the water column. The present study should therefore be regarded as comparative hazard identification under controlled laboratory conditions rather than a quantitative assessment of ecological risk or safety thresholds in natural aquatic environments.

Experimental replication and statistical power also represent important limitations when interpreting the findings. Each treatment included three independent tanks, with the tank serving as the experimental unit. Individual fish, multiple hearts within pooled samples, multiple sections from the same animal, and technical replicates were all treated as subsamples within the corresponding tank. For body length and body weight, the mean of 10 fish from each tank was used for statistical analysis. For biochemical assays and qPCR, multiple hearts from each tank were pooled to generate one sample. Thus, the number of independent biological replicates for each endpoint was three. This design avoided treating subsamples as independent replicates, but with only three independent tanks per treatment, the study had limited power to detect small-to-moderate effects, and confidence intervals around tank-level estimates were necessarily wide. Consequently, nonsignificant comparisons may reflect insufficient power and remain susceptible to Type II error rather than demonstrating the absence of a biological effect. In addition, the present study examined only one polymer type, one nominal particle size, two exposure concentrations, one accelerated aging protocol, and adult male zebrafish. Measured exposure concentrations, tissue particle burdens, leachate controls and blinded histopathological scoring were also lacking. Together, these limitations restrict the extent to which the findings can be generalized.

Zebrafish share several conserved features with other vertebrates in cardiovascular structure, oxidative stress responses, and injury repair mechanisms. Comparative genomic analyses indicate that approximately 70% of human genes have at least one zebrafish ortholog and that approximately 82% of human-disease-related genes have corresponding genes in zebrafish [15]. These characteristics make zebrafish useful for identifying potential cardiac hazards and generating testable mechanistic hypotheses [15,16,17,18,47]. Human studies have detected microplastics or nanoplastics in blood, blood vessels, thrombi, and atherosclerotic plaques and have reported associations with cardiovascular outcomes [2,3,4,5,14]. Against this background, the present comparison of unaged and photoaged PE-MPs in adult zebrafish helps connect material aging characteristics with cardiac oxidative injury, inflammation-related alterations, and extracellular matrix remodeling at the whole-animal level and may help identify biological endpoints and candidate mechanisms that warrant further investigation in human-relevant research. In particular, the redox imbalance, inflammation-related responses, and fibrosis-like remodeling observed here may provide an experimental basis for selecting endpoints in mammalian models, human-derived cardiovascular cell systems, and population studies. The findings also suggest that human health research should not focus solely on unaged particles but should consider how environmental aging may modify the biological effects of microplastics. Nevertheless, waterborne exposure concentrations in zebrafish cannot be directly converted into internal exposure doses in humans, and the present results do not demonstrate that the same pathological processes necessarily occur in humans.

Overall, under the controlled conditions used in this study, PE-MPs induced cardiac oxidative injury, inflammation-related alterations, and fibrosis-like remodeling in adult zebrafish, while photoaging enhanced some, but not all, of these endpoints. Future studies should incorporate exposure concentrations closer to environmentally relevant levels and verified by direct measurement, together with mammalian and human-derived cell models, protein-level analyses, and pathway intervention experiments, to further test the proposed working model and its potential relevance to human cardiovascular health.

## 5. Conclusions

This study demonstrated that UV photoaging increased the surface roughness of PE-MPs, generated more pores and cracks, and enhanced surface-oxidation-related features. Under controlled laboratory conditions, PE-MP exposure disrupted cardiac redox homeostasis in adult zebrafish and was accompanied by inflammation-related histomorphological alterations, increased collagen deposition, and abnormal expression of genes associated with extracellular matrix remodeling. Compared with unaged PE-MPs, photoaged PE-MPs produced more pronounced effects on selected oxidative stress, collagen deposition, and matrix remodeling endpoints, but did not consistently enhance all toxicity endpoints, including growth and body weight. These findings indicate that the influence of photoaging is endpoint-specific.

Taken together, the material characterization, biochemical, histological, and molecular findings support the conclusion that PE-MPs can induce cardiac oxidative injury and fibrosis-like remodeling in adult zebrafish. The results also provide associative support for a conceptual working framework linking material surface aging, redox imbalance, inflammation-related alterations, and extracellular matrix remodeling, but do not establish a continuous causal pathway. Because the nominal exposure concentrations used in this study were higher than most environmentally measured levels, and neither actual exposure concentrations nor aging-derived leachates were directly measured, the findings should be interpreted as evidence from comparative hazard identification under controlled laboratory conditions. They should not be used directly for quantitative assessment of ecological risk in natural environments, human cardiovascular risk, or confirmation of established myocardial fibrosis. Further studies incorporating environmentally relevant exposure conditions, protein-level validation, pathway intervention, and mammalian and human-derived models are needed to clarify the underlying mechanisms and their potential relevance to human health.

## Figures and Tables

**Table 1:** Replicate-level mortality at 96 h in the acute toxicity test.

PE-MP Concentration (mg/L)	Biological Replicates (n)	Zebrafish per Replicate (Total)	Cumulative Deaths at 96 h (R1/R2/R3)	Mean Cumulative Mortality (%)
0	3	10 (30)	0/0/0	0.0 ± 0.0
400	3	10 (30)	1/1/1	10.0 ± 0.0
800	3	10 (30)	5/5/5	50.0 ± 0.0
1200	3	10 (30)	6/7/8	70.0 ± 10.0
1600	3	10 (30)	10/10/10	100.0 ± 0.0
2000	3	10 (30)	10/10/10	100.0 ± 0.0

Note: Values are based on n = 3 independent exposure units per concentration, with 10 zebrafish per unit (30 fish per concentration). The LC_50_ estimated by probit regression was 787.1 mg/L (95% CI: 683.2–890.3 mg/L).

**Table 2:** Apparent oxidation indices of PE-MPs before and after photoaging.

Indicator	Calculation Method	U-MPs	Aged-MPs	Percentage Change
Apparent Carbonyl Index	A1722/A1468	0.133	0.330	+148.4%
Apparent Hydroxyl Index	A3409/A1468	0.140	0.198	+42.2%

Note: Absorbance was converted from transmittance according to A = −log_10_(T/100).

**Table 3:** Collagen-positive area (%) in adult zebrafish hearts after 28 d of PE-MP exposure.

Staining Method	Ctrl	U-MPs-L	U-MPs-H	Aged-MPs-L	Aged-MPs-H
Sirius Red	22.38 ± 1.19	22.51 ± 1.18	24.06 ± 1.32	30.01 ± 1.74	47.60 ± 3.10
Masson	20.73 ± 1.14	21.83 ± 1.20	25.80 ± 1.54	29.45 ± 1.75	36.12 ± 2.20

Note: Data are presented as mean ± SD; n = 3 independent tanks per treatment group. The unit of collagen-positive area is %.

## Data Availability

The original contributions presented in this study are included in the article/Supplementary Materials. Further inquiries can be directed to the corresponding authors.

## Supplementary Materials

The following supporting information can be downloaded at: https://www.mdpi.com/article/10.3390/toxics14100889/s1, Table S1: Rearing Conditions and Others; Table S2: Primer sequences used for quantitative real-time PCR.

## Abbreviations

The following abbreviations are used in this manuscript:

CAT	Catalase
Ctrl	Control
FTIR	Fourier transform infrared spectroscopy
HE	Hematoxylin and eosin
LC50	Median lethal concentration
MDA	Malondialdehyde
PE-MPs	Polyethylene microplastics
qPCR	Quantitative real-time polymerase chain reaction
SEM	Scanning electron microscopy
T-SOD	Total superoxide dismutase
U-MPs	Pristine (unaged) polyethylene microplastics
