# Supplementary material for: Effects of Photoaged Polyethylene Microplastics on Cardiac Injury and Fibrosis-like Remodeling in Zebrafish
Source: Toxics. 2026 Oct 5;14(10):889. doi: 10.3390/toxics14100889 (PMC13709661; doi:10.3390/toxics14100889)
Supplement: Supplementary file 1 [file toxics-14-00889-s001.zip › toxics-4566227-SI.pdf]

Table S1. **Rearing Conditions and Others**

**Water quality**	Temperature	28.0 ± 0.5 °C	-	Controlled by the aquarium heating system
	pH	7.1 ± 0.2	-	Measured daily
	Photoperiod	14 h light/10 h dark	-	Controlled by an automatic timer
**Routine diet**	Feed	Shrimp eggs	Commercial supplier	One dose twice daily
**Microplastic**	Polyethylene (PE)	Approx. 50 μm particle size, spherical, >99% purity	Taisulang Chemical Materials Co., Ltd. (Dongguan, China)	No fluorescent labeling
**Other reagents**	Tricaine (anesthetic)	0.02% solution	Sigma-Aldrich (USA) / [Catalog No.]	Used for immobilization prior to euthanasia
**Culture medium**	E3 solution	**50× stock solution:**<br>- Sodium chloride: 72.5 g/5 L<br>- Potassium chloride: 3.4 g/5 L<br>- Calcium chloride: 9.2 g/5 L<br>- Magnesium sulfate heptahydrate: 40 g/5 L<br>**1× working solution:**<br>- 50× stock solution: 100 mL<br>- Purified water: 5 L<br>- Methylene blue: 1.0 mL	Prepared in-house using analytical-grade reagents	Standard formulation for zebrafish culture
**Test organism**	Zebrafish (Danio rerio)	AB wild type, adult males, 4-5 months old	Shandong Yixuyue Biotechnology Co., Ltd. (China)	Stocking density: <5 fish/L

Table S2. Primer sequences used for quantitative real-time PCR.

Gene	Forward primer (5′–3′)	Reverse primer (5′–3′)	Expected amplicon size (bp)	Annealing/extension temperature (°C)
il1b	GTCACACTGAGAGCCGGAAG	GCAGGCCAGGTACAGGTTAC	135	60
acta2	AAGGAGATCACTGCTTTGGC	CTTGCGATGCACAATAGAGG	177	60
ccn2a	GTAACCGTCGTATTGGTGTCTGC	GCACTTCCCAGGCACTTTCA	218	60
col1a1a	CAACCCAACTGAGTCTGCTATTCC	CAGGAAGGTGAGCTGAATGTTGA	166	60
mmp9	TAGCGGATGAGTATCTGAAGCG	GAATGTCTTGTAGTTGCGGATGT	206	60
eef1a1l1	TGAAGACAACCCCAAGGCTC	GTCCTTAAGTAGAGTGCCCAGG	319	60

Note: All primer sequences are shown in the 5′–3′ direction. *eef1a1l1* was used as the reference gene. The temperature shown is the PCR annealing/extension temperature and should not be interpreted as the theoretical melting temperature of each primer.
